# Computational Modeling Reveals Distinct Effects of HIV and History of Drug Use on Decision-Making Processes in Women

**DOI:** 10.1371/journal.pone.0068962

**Published:** 2013-08-07

**Authors:** Jasmin Vassileva, Woo-Young Ahn, Kathleen M. Weber, Jerome R. Busemeyer, Julie C. Stout, Raul Gonzalez, Mardge H. Cohen

**Affiliations:** 1 Department of Psychiatry, University of Illinois at Chicago, Chicago, Illinois, United States of America; 2 Virginia Tech Carilion Research Institute, Roanoke, Virginia, United States of America; 3 Core Center/Cook County Health & Hospital Systems, Chicago, Chicago, Illinois, United States of America; 4 Department of Psychology, Indiana University, Bloomington, Indiana, United States of America; 5 School of Psychology and Psychiatry, Monash University, Clayton Campus, Victoria, Australia; 6 Department of Psychology, Florida International University, Miami, FL, United States of America; 7 Department of Medicine, Rush University Medical Center, Chicago, Illinois, United States of America; Centre national de la recherche scientifique, France

## Abstract

**Objective:**

Drug users and HIV-seropositive individuals often show deficits in decision-making; however the nature of these deficits is not well understood. Recent studies have employed computational modeling approaches to disentangle the psychological processes involved in decision-making. Although such approaches have been used successfully with a number of clinical groups including drug users, no study to date has used computational modeling to examine the effects of HIV on decision-making. In this study, we use this approach to investigate the effects of HIV and drug use on decision-making processes in women, who remain a relatively understudied population.

**Method:**

Fifty-seven women enrolled in the Women's Interagency HIV Study (WIHS) were classified into one of four groups based on their HIV status and history of crack cocaine and/or heroin drug use (DU): HIV+/DU+ (n = 14); HIV+/DU− (n = 17); HIV−/DU+ (n = 14); and HIV−/DU− (n = 12). We measured decision-making with the Iowa Gambling Task (IGT) and examined behavioral performance and model parameters derived from the best-fitting computational model of the IGT.

**Results:**

Although groups showed similar behavioral performance, HIV and DU exhibited differential relationship to model parameters. Specifically, DU was associated with compromised learning/memory and reduced loss aversion, whereas HIV was associated with reduced loss aversion, but was not related to other model parameters.

**Conclusions:**

Results reveal that HIV and DU have differential associations with distinct decision-making processes in women. This study contributes to a growing line of literature which shows that different psychological processes may underlie similar behavioral performance in various clinical groups and may be associated with distinct functional outcomes.

## Introduction

HIV and drug addiction are truly linked epidemics [1] with known effects on fronto-striatal systems and associated impairments in executive cognitive functions [2]–[4]. Among the latter, decision-making is often prominently affected in HIV-seropositive (HIV+) [5], [6] and HIV-seronegative (HIV−) drug users [7]–[10].

“Decision-making” is typically defined as the ability to select advantageously from an array of available options, such that response selections result in long-term positive outcomes. It is often studied using laboratory tasks such as the Iowa Gambling Task (IGT), which simulates real-life situations in the way it involves uncertainty, reward, and punishment [11]. The IGT was developed originally to capture deficits in decision-making among persons with focal lesions of the ventromedial prefrontal cortex [11], [12] who displayed seeming indifference to the long-term consequences of their actions, as evidenced by excessive choices of immediately attractive but ultimately disadvantageous outcomes. Substance dependent individuals typically show impaired performance on this task [7], [8], [13]–[16]. Recently, decision-making has received increased attention in the HIV literature [5], [6], [17], [18], in large part because of its association with behaviors that increase risk for HIV infection and transmission [19].

The IGT is a complex task and poor behavioral performance could be the result of deficits in various distinct component processes, such as hypersensitivity to reward and/or hyposensitivity to losses, failure to learn from past outcomes and losses, and/or erratic and impulsive response style [20], [21]. In a series of studies, Busemeyer, Stout and their colleagues [20], [22], [23] have developed mathematical models of the task that capture the complex interplay of cognitive and motivational processes involved in decision-making. The use of such models allows one to decompose IGT behavioral performance into distinct cognitive, motivational, and response processes, thereby providing a fine-grained analysis of the underlying decision-making processes and characterizing more precisely the decision-making deficits of different clinical groups. Computational modeling has been used successfully to investigate components of impaired IGT performance among drug users [20], [24], [25], incarcerated criminal offenders [21], patients with bipolar disorder [26], schizophrenia [27], autism spectrum disorders [28], and Huntington's disease [29]. Studies applying this approach show that although behavioral performance may be similar across different clinical groups, the cognitive processes that underlie these behavioral profiles may vary across groups in clinically meaningful ways.

To our knowledge, no studies to date have investigated the IGT performance of HIV-seropositive individuals using the computational modeling approach, despite evidence that HIV has detrimental effects on executive function and decision-making which have become even more pronounced since the advent of combination highly active antiretroviral therapy (cART) [3]. There is evidence that male HIV+ drug users perform the IGT significantly more poorly (i.e., make significantly more disadvantageous card selections) compared with demographically matched HIV− drug users [6]. Similarly, Hardy et al. [5] and Thames et al. [18] revealed that a gender mixed group (34% women in [5] and 24% women in [18]) of HIV+ drug users and non-drug users made significantly more disadvantageous choices on the IGT than HIV− controls. Yet, neither one of these studies reported IGT results separately by gender nor did they control for gender in their analyses. Overall, no studies to date have investigated the integrity of decision-making mechanisms in HIV+ women as a function of HIV and drug use. This is not entirely surprising, given that women have been generally under-represented in neuropsychological studies of HIV [30]. Yet, HIV+ women may be at greater risk for cognitive decline than HIV+ men [30]–[32] and there are known gender differences in decision-making on the IGT, with males typically outperforming females on the task [11], [33], [34].

In the current investigation, we tested three computational models of the IGT in a sample of HIV+ and HIV− women with and without a history of crack cocaine and/or heroin use. We evaluated both the standard behavioral performance scores from the IGT, as well as model parameters derived from the best-fitting computational model, in order to determine if those differ systematically according to HIV serostatus and/or history of drug use. We hypothesized that HIV+ women with a history of drug use would be most impaired on the task and that the performance of HIV− women with no history of drug use would be impaired least. In line with the literature [20], [23]–[25], we hypothesized that a history of drug use would be related to loss aversion and reward sensitivity, with the caveat that more specific predictions were precluded due to the relatively limited literature with women on decision-making. For similar reasons, we did not make more specific predictions regarding how HIV might affect specific IGT components, although we expected that HIV+ women would evidence impaired performance on the task. Finally, in order to follow up on our earlier finding that performance on the IGT may relate to risky behaviors [19] and to examine the relationships between model parameters and functional outcomes, we evaluated the associations between component processes of the IGT and risky sexual practices and with how closely HIV+ participants followed their recommended medication schedule. We hypothesized that high sensitivity to reward and/or reduced sensitivity to loss would be related to more risky behaviors and poorer adherence to HIV medication schedules and that this effect would be more pronounced in HIV+ women with a history of drug use.

## Methods

### Ethics Statement

The study was conducted according to the principles expressed in the Declaration of Helsinki. The study protocol was approved by the Institutional Review Boards at University of Illinois at Chicago and the Cook County Health and Hospitals System. All enrolled participants gave written informed consent for participating in the study.

### Participants

Fifty-seven women were recruited from the Chicago site of the Women's Interagency HIV Study (WIHS), the largest ongoing longitudinal study of HIV disease in women in the United States. The WIHS is a multi-center study of HIV seropositive and HIV seronegative women established in 1994. The HIV+ and HIV− cohorts were recruited from similar sources and were matched on demographics and key risk factors, such as age, race/ethnicity, education, injection drug use, and number of sexual partners, as described in Barkan et al. [35] and Bacon et al. [36]. The WIHS protocol includes a baseline visit and follow-up visits on a semiannual basis, which include a physical examination, collection of blood for biomarker measurement, and completion of various questionnaires and tasks. Participants were recruited for this sub-study during their semiannual WIHS visits at the CORE Center at Cook County Health and Hospital Systems, by prior review of WIHS variables to pre-identify women for each group, who were then approached and the ones who consented were enrolled sequentially into the current sub-study. HIV serostatus was verified by repeatedly reactive enzyme-linked immunosorbent assay (ELISA) and confirmed by Western Blot. Women whose primary first language was not English, who had a history of closed head injury with loss of consciousness exceeding 30 minutes, open head injury, psychotic disorders, or current neuroleptic use were excluded from participation.

The majority of the participants (81%) were African-American, 14% were Hispanic, and 5% were white. Thirty-one of the women were HIV-seropositive (HIV+) and 26 were HIV-seronegative (HIV−). Further, 28 of the women had a history of crack cocaine and/or heroin use (DU+) and 29 had no such history (DU−). Overall, 14 participants were HIV+/DU+, 17 were HIV+/DU−, 14 were HIV−/DU+, and 12 were HIV−/DU− (see Tables 1 and 2).

**Table 1:** Demographic and HIV disease characteristics of participants.

	HIV+/DU+ (n = 14)	HIV+/DU− (n = 17)	HIV−/DU+ (n = 14)	HIV−/DU− (n = 12)	*p*
Age (SD)	43.3 (4.9)	38.8 (8.3)	40.6 (7.1)	33.5(8.5)	*p* = .01
Education (SD)	11.3 (1.01)	10.9 (2.1)	11.5 (.73)	11.7 (.49)	*p* = .51
*Race (%)*					*p* = .43
African-American	86	71	93	75	
Hispanic	7	23	0	25	
Caucasian	7	6	7	0	
WTAR Reading	27.3 (5.1)	29.8 (11.4)	25.2 (10.1)	28.6 (10.6)	*p* = .59
*cART*					*p* = .90
Currently on cART (%)	86	88	-	-	
Not on cART (%)	14	12	-	-	
CD4 count at closest WIHS visit	428.07 (273.9)	481.6 (245.6)	-	-	*p* = .57
Nadir CD4 count	324.4 (174.1)	288.1 (85.9)	-	-	*p* = .47

Note: Unless otherwise stated, data are presented as means and standard deviations.

**Table 2:** Substance use characteristics of participants.

	HIV+/DU+ (n = 14)	HIV+/DU− (n = 17)	HIV−/DU+ (n = 14)	HIV−/DU− (n = 12)	*p*
***KMSK Total Scores***					
Alcohol	8.43	6.59	7.79	8.0	*p* = .56
Tobacco	10.21	2.53	9.64	5.17	*p*<.0001
Cocaine	13.43	.12	10.07	0	*p*<.0001
Heroin	3.07	0	5.21	0	*p*<.0001
***KMSK lifetime heroin use***					
Never used (%)	9 (64)	17 (100)	6 (43)	12 (100)	
20–100 times/lifetime (%)	1 (7)	0	1 (7)	0	
>100 times/lifetime (%)	4 (29)	0	7 (50)	0	
***KMSK lifetime cocaine use***					
Never used (%)	1 (7)	16 (94)	3 (22)	12 (100)	
Fewer than 20 times/lifetime (%)	0	1 (6)	1 (7)	0	
20–100 times/lifetime (%)	0	0	1 (7)	0	
>100 times/lifetime (%)	13 (93)	0	9 (64)	0	
***KMSK Current Use***					
Cocaine (%)	1 (7)	0	2 (14)	0	
Heroin (%)	0	0	2 (14)	0	
***Marijuana Use***					
Proportion of WIHS visits reporting marijuana use	.13 (.23)	.24 (.38)	.28 (.34)	.35 (.39)	*p = .42*

### Assessment procedures

#### Intellectual Functioning

Intellectual functioning was estimated with the Wechsler Test of Adult Reading (WTAR™) [37].

#### Substance Use

Substance use was quantified by the Kreek-McHugh-Schluger-Kellogg Scale (KMSK) [38], a brief screening instrument which indexes the degree of self-exposure to four different classes of drugs (alcohol, tobacco, cocaine, and heroin) defined as the frequency, amount, and duration of use during the lifetime period of greatest consumption of these substances. The total score for each of the four classes of substances on the KMSK was determined by summing the frequency, duration, and amount of use of each substance. The KMSK shows excellent associations with DSM-IV criteria for substance dependence as measured by the SCID, with very high specificity and sensitivity for opiates (100% and 99% respectively) and cocaine (97% and 94% respectively) [38]. Scores range from 0–12 for tobacco, 0 to 13 for alcohol and for opiates, and from 0 to 16 for cocaine. As recommended by Tang et al. [39] who evaluated the KMSK with poor urban predominantly female African-Americans, a population very similar in demographic characteristics to our participants, subjects were considered as DU+ if they scored a minimum of 6 on the cocaine subscale and/or 2 on the opiates subscale. The range of scores on the cocaine scale of HIV+/DU+ participants who reported cocaine use was between 10 and 16, and between 6 and 16 in HIV−/DU+ participants reporting cocaine use. On the opiates scale, scores of HIV+/DU+ participants who reported opiate use ranged between 6 and 11 and scores of HIV−/DU+ participants reporting opiate use ranged between 7 and 13 (see also Table 2). Given that the KMSK scale does not assess marijuana use, we did not have a formal measure of severity of marijuana use. However, we had available information on proportion of WIHS visits at which subjects reported marijuana use within the previous 6 months, which we used as an index of marijuana use (Table 2).

#### HIV Risk Behaviors and Adherence to HIV Medication Schedule

We administered the Risk Assessment Battery (RAB), to measure HIV risk behaviors during the past 6 months [40]. It is comprised of two subscales: “Needle Use” (RAB-NU) and “Sexual Practices” (RAB-SP) reflecting frequency or quantity of specific behaviors that a participant may have engaged in during the past 6 months. Most participants in our sample were not actively using injection drugs during the 6 months prior to their assessment; therefore, the variability of scores on the RAB-NU subscale was extremely restricted, median = 0, IQR [0, 0]. For the RAB-SP subscale, our sample displayed ample variability (range = [0, 13], median = 4, IQR [2], [5]). Given that essentially all of the variance in the RAB total scores was the result of responses on the RAB-SP subscale, we only used the RAB-SP in the statistical analyses.

Degree of adherence to antiretroviral therapy among HIV+ participants was evaluated by asking participants at their nearest WIHS core visit how closely they have followed their HIV medication schedule during the past six months. Responses were coded as follows: 1 = never; 2 = some of the time; 3 = about ½ of the time; 4 = most of the time; 5 = all of the time.

#### Decision-Making

Participants were administered a computerized version of the Iowa Gambling Task (IGT) [7]. The task requires participants to make selections from four decks of cards (A, B, C, and D) with the goal of maximizing profit on a loan of play money. Participants are instructed to select one card on each trial and are told that each card selection would be associated with a win of some money; however, occasionally, a particular card selection would also result in a loss. Decks A and B are “disadvantageous” in that they are associated with high immediate rewards but even higher subsequent losses and therefore are more costly in the long run. Decks C and D, on the other hand, are considered “advantageous” because they result in an overall long-term gain. Ultimately, selecting from the “good decks” results in a net profit at the end of the task, whereas selecting from the “bad decks” results in a net loss. Importantly, participants are not told that the different decks are associated with differential schedules of rewards and punishments and they have to learn the task contingencies by trial-and-error as the task progresses. Participants are given visual and auditory feedback about their gains and losses after each card selection. Normal healthy adults typically learn which decks are advantageous for maximizing monetary gain, as indicated by their increasing proportion of choices from the advantageous decks.

The task consists of 100 trials. Each deck of cards has 60 cards and if the cards ran out from a deck, subjects had to choose from other decks thereafter. If one of the decks was depleted during the task (i.e., chosen 60 times), the remaining trials were not used for the computational modeling analysis because the structure of the task at that point becomes a choice among 3 decks instead of 4 decks. Unlike the original IGT [11], the amount of gains are not fixed to $100 or $50, but decks pay an average of $100 and $50. In addition, the amounts of payoffs in all four decks increase across each block of ten cards. See Bechara et al. [7] for more details on the modifications.

### Computational Modeling Analysis of IGT

To determine which computational model best fit our data, we compared three models: (1) the “classic” Expectancy Valence Learning (EVL) model [22]; (2) the Prospect Valence Learning (PVL) model [41] with the delta learning rule [42]; and (3) the PVL model with the decay-reinforcement learning rule [43]. The PVL model with the decay-reinforcement learning rule had the best model-fits (see Appendix S1 in File S1 for details of the model comparisons).

The outcome evaluation in the PVL model follows the *prospect utility function* which has diminishing sensitivity to increases in magnitude and differential sensitivity to losses versus gains. The PVL model has four parameters (see Appendix S2 in File S1 for mathematical details of the model): (1) the *shape of the utility function* parameter ***α*** (0<*α*<1), which reflects ***reward sensitivity***. As this parameter approaches 1 (i.e., high reward sensitivity), the subjective utilities of outcomes increase in direct proportion to the actual outcomes, but as the parameter approaches 0 (i.e., low reward sensitivity), the subjective utilities increase non-linearly in a step-wise fashion, such that all gains and all losses are subjectively equal; (2) the ***loss aversion parameter λ***, (0<*λ*<5), which determines the sensitivity to losses compared to gains and reflects the tendency to select the alternative that decreases the probability of losses even if it is associated with lower expected gains (Ahn et al. 2008). A value of loss aversion (*λ*) greater than 1 indicates that the individual is more sensitive to losses than to gains. Conversely, a value of *λ* less than 1 indicates that the individual is more sensitive to gains than to losses; (3) the ***recency parameter A*** (0<*A*<1), which determines how much the past expectancy is discounted. A high value of *A* indicates good learning/less memory decay, whereas a low value of A indicates rapid memory decay; (4) the ***consistency parameter (c)***, which indicates how close the decision-maker's selections adhere to their expectancies of the decks' utilities. Consistency (*c*) values range between 0 and 5, which allows for totally random and presumably impulsive choices (*c* = 0) to almost deterministic choices (*c* = 5). The softmax choice rule [44] was used to compute the probability of choosing each deck.

In sum, the PVL model has four free parameters that reflect distinct psychological processes: (1) *α*, utility shape (reward sensitivity); (2) *λ*, loss aversion; (3) *A*, recency (learning/memory); and (4) *c*, choice consistency. We used hierarchical Bayesian analysis (HBA) for estimating model parameters (see Appendix S3 in File S1 for details). HBA has several advantages over null hypothesis significance testing (NHST) and provides more robust and stable estimation of individual and group differences (see [45] for review). We evaluated group differences on model parameters estimated from hierarchical Bayesian analysis by examining 95% highest density interval (HDI), which is an interval that spans 95% of the posterior distribution [46]. For example, if 95% HDI of group differences excludes zero, this would indicate that the groups are *credibly* different. For clarity, we also report in parallel NHST results.

## Results

### Demographic, Substance Use, and HIV Disease Characteristics of Participants

Group differences in continuous variables were examined using one way ANOVAs with Tukey HSD post hoc comparisons where appropriate. Categorical variables were examined using Pearson's chi square analyses. Groups were well matched on education, ethnicity, and estimated premorbid intelligence (Table 1). There were significant group differences in age (*F*_(3,53)_ = 4.05, *p* = .01), with the HIV+/DU+ group being older than the HIV−/DU− group. Among HIV+ participants, there were no group differences between the DU+ and DU− groups on current CD4 count (*F*_(1,29)_ = .33, *p* = .57), nadir CD4 (*F*_(1,29)_ = .54, *p* = .47), or how closely they followed their recommended HIV medication dosing schedule (*χ* ^2^_(3)_ = 2.7, *p* = .44). Eighty-six percent of HIV+/DU+ participants were currently on cART, and 14% were not on any antiretroviral therapy. Among HIV+/DU− participants, 88% were currently on cART and 12% were not taking any HIV medication. The two DU+ groups were also well matched on substance use characteristics as measured by the KMSK (Table 2). Specifically, there were no significant group differences in total cocaine scores (*F*_(1,26)_ = 2 .73, *p* = .11), heroin scores (*F*_(1,26)_ = 1.48, *p* = .24), tobacco scores (*F*_(1,26)_ = 0.31, *p* = .59) and alcohol scores (*F*_(1,26)_ = 0.19, *p* = .67) between HIV+/DU+ and HIV−/DU+ participants. The two DU+ groups scored significantly higher than the two DU− groups in total cocaine scores (*F*_(3,53)_ = 45 .78, *p*<.0001), heroin scores (*F*_(3,53)_ = 8.65, *p*<.0001) and tobacco scores (*F*_(3,53)_ = 18.59, *p*<.001), but not in alcohol scores (*F*_(3,53)_ = .70, *p* = .56).

### Behavioral Performance on the Iowa Gambling Task

**Figure 1:**
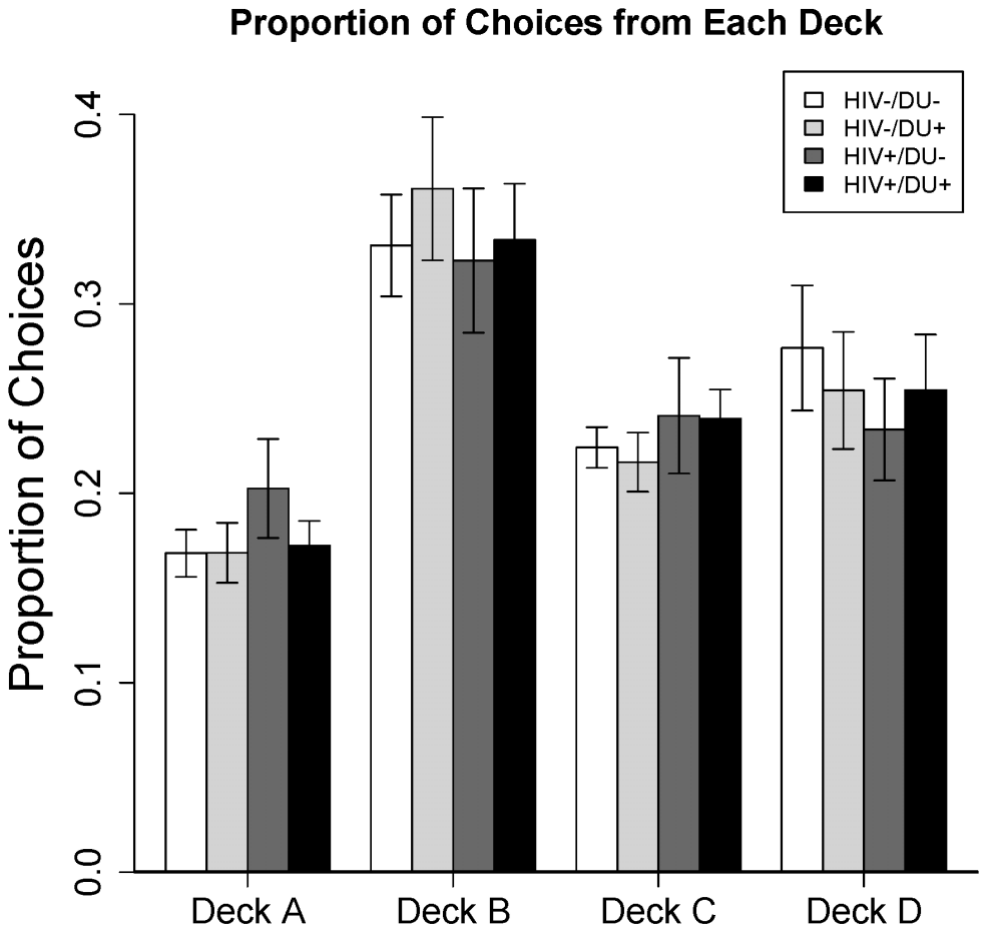
IGT performance (overall proportion of choices from each deck). **Error bars indicate ±1 SEM.**

IGT performance was analyzed with a mixed model ANOVA with trial block (Blocks 1–5) as the within subject factor, and HIV (HIV+, HIV−) and drug use (DU+, DU−) as the between-subjects factors. When sphericity could not be assumed, Greenhouse-Geisser correction was applied. The analysis revealed no significant main effects or interactions (all *p*'s>.1). In general, all four groups made more disadvantageous than advantageous selections throughout the task, as indicated by their predominantly negative net scores (HIV+/DU+: −1.14 (5.8); HIV+/DU−: −4.4 (6.5); HIV−/DU+: −5.9 (6.8); HIV−/DU−: .17 (5.2). Further, none of the groups learned to switch their selections from the disadvantageous to the advantageous decks as the task progressed, indicated by the lack of significant main effect of trial block (*F*_(4,212)_ = 1.11, *p* = .35). There were also no group differences in preferences for specific decks throughout the task, with participants across all four groups making more choices from deck B than from any of the other decks (*F*_(2.29,121.1)_ = 17.26, *p*<.001, with Greenhouse-Geisser correction for violation of sphericity applied). Eighteen percent of participants' choices were from Deck A, 34% of choices - from Deck B, 23% - from Deck C, and 25% - from Deck D (Figure 1).

### Computational Modeling Results

**Figure 2:**
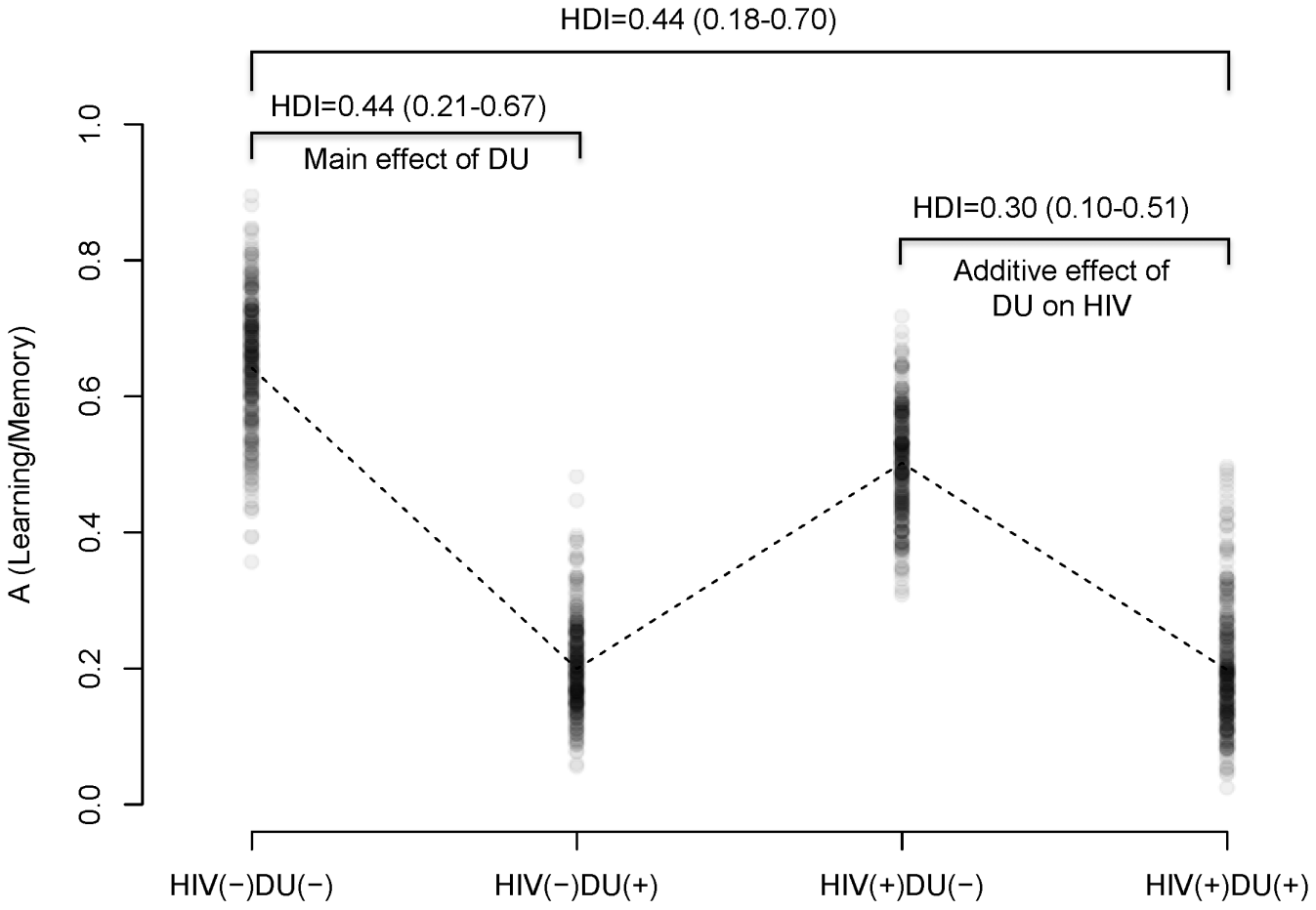
Parameter estimates of A (learning/memory). Note: 300 random samples were drawn from the posterior distributions for each group. Dashed lines indicate mean values for each group. HDI = mean and 95% HDI range.

**Figure 3:**
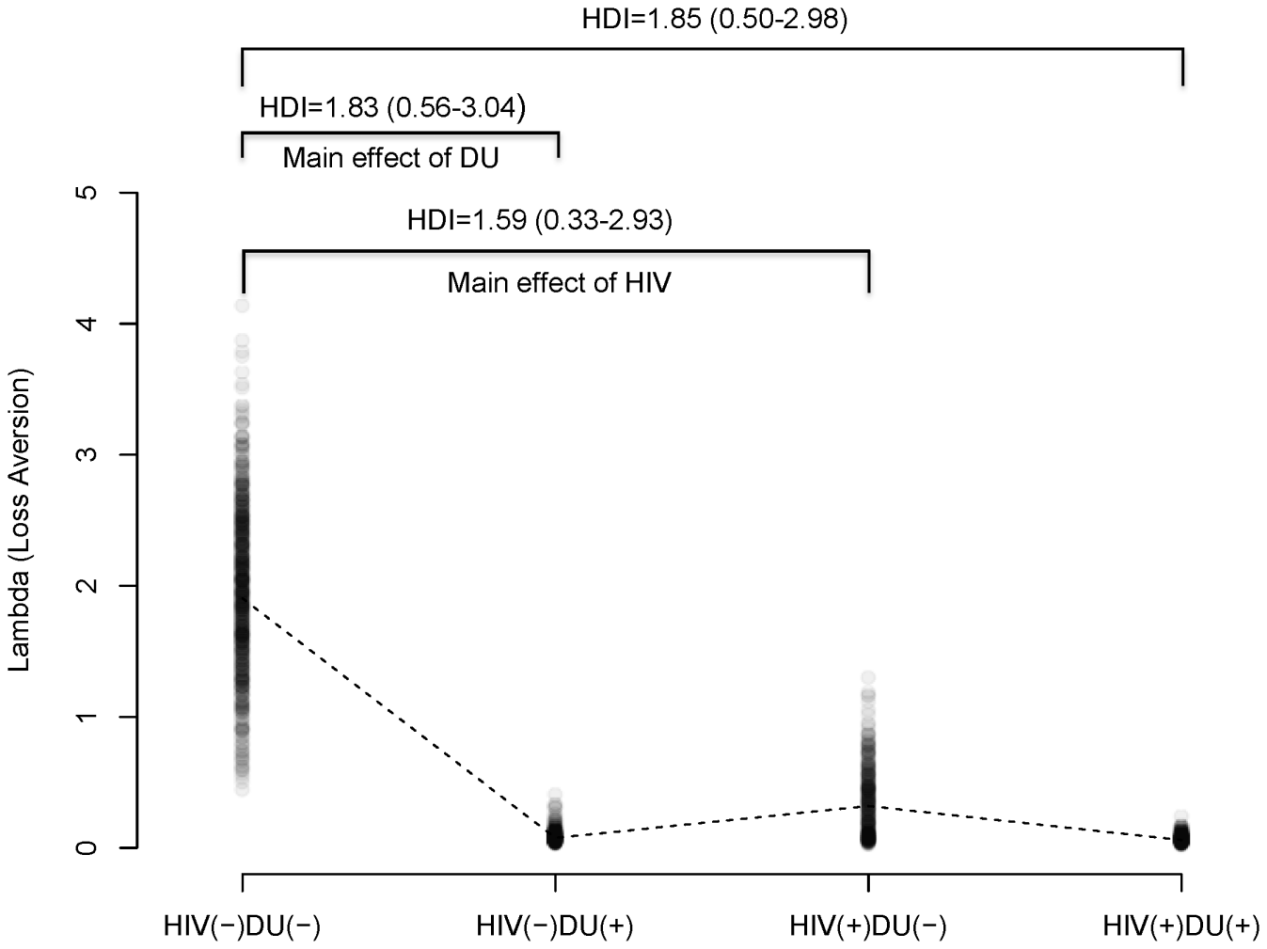
Parameter estimates of λ (loss aversion). Note: 300 random samples were drawn from the posterior distributions for each group. Dashed lines indicate mean values for each group. HDI = mean and 95% HDI range.

We next compared the four groups on the parameter estimates from the PVL model. Table 3 summarizes PVL model parameter estimates from the HBA. We examined whether HIV or DU affect model parameters by comparing the control group (HIV−/DU−) with the groups with single risk factor (HIV+/DU− and HIV−/DU+) (i.e. “main effects” of HIV or DU). Among the four parameters, we found that DU was associated with the learning/memory parameter *A* (Figure 2) and loss aversion _ (Figure 3), while HIV was related only on loss aversion (Figure 3). HIV−/DU+ participants had compromised learning/memory function indicated by a reduced recency parameter (Figure 2) and lower loss aversion than HIV−/DU− control participants (mean difference = .44, 95% HDI of group differences from .18 and .70; NHST: t(24) = 8.86, *p*<.0001, see also Figure 3). On the other hand, HIV+DU− participants were less averse to loss compared to HIV−/DU− participants (mean difference = 1.59, 95% HDI of group differences from .33 and 2.93; NHST: t(27) = 8.54, *p*<.0001).

**Table 3:** Means and (standard deviations) of computational modeling parameters.

	HIV+DU+	HIV+DU−	HIV−DU+	HIV−DU−
**Recency (** ***A*** **)**	.20 (.04)	.50 (.19)	.21 (.06)	.64 (.18)
**Reward Sensitivity (** ***α*** **)**	.38 (.04)	.26 (.02)	.36 (.03)	.18 (.02)
**Consistency (** ***c*** **)**	.49 (.40)	.55 (.38)	.62 (.38)	.36 (.30)
**Loss Aversion (** ***λ*** **)**	.06 (.01)	.25 (.16)	.07 (.01)	1.84 (.75)

Given the significant group differences in age, we conducted additional Bayesian and NHST analyses controlling for age, which revealed essentially identical findings. See Appendix S3 in File S1 for details of the Bayesian analysis controlling for age.

### Associations of Modeling Parameters with HIV Risk Behaviors and HIV Medication Adherence

Bivariate correlational analyses, conducted separately for each of the four groups explored associations between the four computational modeling parameters on the IGT and HIV risky sexual behaviors as measured by the RAB-SP. Significant associations with RAB-SP were observed only in the HIV+/DU+ group, where it was related to reward sensitivity (r = .59, *p* = .02) and learning/memory (r = .58, *p* = .03). No significant correlations between modeling parameters and the RAB-SP were observed in the other groups.

Additional correlational analyses restricted to the two HIV+ groups explored the associations between modeling parameters and how closely participants followed their recommended HIV medication schedule. Again, a significant correlation was observed only within the HIV+/DU+ group between consistency (*c*) and degree of medication adherence (*r_s_* = .71, *p*<.01), whereas no significant associations were noted in the HIV+/DU− group.

## Discussion

To our knowledge, this is the first study that employs computational modeling to investigate the effects of HIV and history of drug use on decision-making. Results from these analyses indicate that even within the context of no differences in overt behavioral performance, there are notable differences in the underlying components of decision-making that appear to be differentially related to HIV and drug use. Specifically, we found that whereas both HIV and DU were associated with lower loss aversion, DU was additionally related to compromised learning/memory. Importantly, results revealed that some of the cognitive and motivational processes involved in decision-making may have functional significance for HIV infected women with a history of illicit drug use (HIV+/DU+), among which some of the parameters were related to risky sexual behaviors and reduced adherence to HIV medication dosing schedules.

Although deficits in decision-making are a common finding in the addiction literature [7], [8], the underlying nature of these deficits has only recently begun to be investigated. The current state of our knowledge about decision-making processes in substance dependent HIV infected women is limited. The use of computational models of decision-making allowed us to conduct an in-depth investigation of such processes in women and to demonstrate that both HIV and DU are associated with decision-making biases related to relative insensitivity to losses. Given that to our knowledge, no studies have applied the computational modeling approach to HIV, the association of HIV with loss aversion that we observed needs to be replicated and explored in larger and mixed gender studies. On the other hand, our findings on the association of DU with loss aversion is consistent with the literature, which reveals reduced loss aversion among users of different classes of drugs such as cocaine [20], cannabis [24] or alcohol [25] relative to controls.

Computational modeling analyses further revealed that a history of cocaine and/or heroin use was specifically associated with the learning/memory parameter of the IGT, whereas HIV was not significantly related to this component of decision-making. Learning and memory processes play a major role in the IGT, as good performance on the task requires participants to learn by trial-and-error which decks are advantageous and to proceed to select consistently from these decks in order to achieve optimal performance. Learning and memory processes are also centrally involved in drug addiction and are of particular etiological significance for the chronic-relapsing nature of the disease [47]–[50]. The effects of DU on learning/memory that we found are in line with previous studies with drug users [23]–[25], although such effects are not invariably observed (see [20]) and need to be investigated further. Of particular relevance to the current study, Stout and colleagues [23] found that in women, only the learning/memory parameter distinguishes between drug using and control participants, whereas the motivational parameter discriminated between drug using men and controls. Similar sex differences have also been noted in other aspects of the IGT. In terms of behavioral performance, research consistently demonstrates that men typically show better performance on the IGT than women [20], [33], [34], [51]–[55]. There is also evidence that drug-using men perform significantly worse than healthy control men on the task, whereas drug-using women perform significantly better than healthy women [23]. Our study provides the first in depth investigation of the psychological processes involved in complex decision-making in women with HIV and DU. Given that no studies to date have directly compared male and female HIV+ drug users both in terms of overall performance and on component processes of decision-making, future studies should systematically investigate whether the associations that we observed are gender specific.

Contrary to predictions, we found no significant group differences in behavioral performance on the IGT, although performance of all four groups remained overall in the impaired range. This finding stands in contrast to numerous studies reporting that drug users are behaviorally more impaired than controls [7], 8 and the growing literature revealing similar impairments in HIV [5], [6], [18]. However, it should be noted that drug users are not invariably impaired on the IGT, as evidenced by studies reporting no group differences between drug users and controls [56], [57]. The lack of group differences in task performance in our study could be related to the distinct nature of our HIV− and DU− control participants, who were recruited and selected from the same high risk population as the HIV+ participants, which is indeed one of the unique aspects of the WIHS parent study. Another somewhat unexpected finding was the absence of learning effects in the performance of all four groups on the IGT. Even though this finding was surprising, it is in line with studies showing that the typical learning effects on the IGT are displayed more commonly by men than by women [51]. The observed inconsistencies in the literature could be due to additional differences in sample characteristics such as age, type of drug(s) used, or length of abstinence. For instance, most of the participants in our study were generally older than participants in previous studies (e.g. [23]–[25]). Further, whereas most previous computational modeling studies have assessed the effects of drug use with current users [9], [20], [23], [24], only about 10% of our DU+ participants were current drug users. In relation to our findings, this suggests that the overt behavioral manifestations of impairments in decision-making may dissipate with abstinence from drug use; however the underlying decision-making biases may remain and may continue to influence important daily activities and risk behaviors. Of note, the learning/memory effect that we observed appears to have a real-world functional significance for the women with co-occurring HIV and history of drug use (HIV+/DU+), as suggested by its relationship with high risk sexual activities on the RAB-SP, which were also associated with increased reward sensitivity in this group. Yet, the association between learning/memory and sexual risk taking was somewhat counterintuitive, in that better learning/memory was associated with *higher* rates of risky sexual behaviors in HIV+/DU+ participants. While these findings need to be explored further, they raise interesting questions regarding decision-making biases and correlates of risky sexual behaviors in HIV+/DU+ women. Further, although high-risk behaviors in drug users have been a subject of intense investigation, most studies have investigated HIV seronegative drug users, whereas the mechanisms driving risky sexual behaviors in HIV seropositive individuals still remain poorly understood. Our results also revealed that a different component of decision-making, namely consistency of responding (*c*) is positively associated with a different type of functional outcome, i.e. degree of adherence to HIV medication schedule. This indicates that an erratic choice pattern or decision-making style may have important negative implications for daily functional behaviors such as medication management, that are critical for suppressing HIV viral replication and disease progression.

This study has several limitations. We did not conduct formal diagnostic interviews for substance use disorders, yet the high correlations between KMSK scores and DSM-IV criteria for substance dependence measured by the SCID increase our confidence in the classification of participants into DU+ and DU− groups. Given that we did not conduct urine toxicology screens, it is possible that not all participants were drug-free at the time of testing. Other caveats are our relatively small sample size and the somewhat non-representative nature of the WIHS cohort, which may limit the generalizability of our findings. Specifically, participants enrolled in the WIHS are consistently offered or referred for services such as HIV counseling, targeted health assessments, health education, treatment for substance abuse, mood disorders, and HIV primary and specialty care [35] that may not be as readily available in the context of standard care. Also worth acknowledging is that the combinations of antiretroviral medications (cART) used by 87% of our HIV+ participants may have affected their neurocognitive functioning. Different cART medications are characterized by varying degrees of CNS penetration effectiveness, which may differentially affect neurocognitive function. The CNS Penetration Effectiveness (CPE) Scale introduced by Letendre and colleagues [58] provides a quantitative index of the relative capacity for an antiretroviral drug to cross the blood brain barrier. However, the relationship between CNS penetration effectiveness of antiretroviral (cART) cocktails and neurocognitive performance is not straightforward, with some studies reporting positive relationships between CPE scores and neurocognitive performance [59]–[61], whereas others report inverse [62], [63] or no relationships [64]. In terms of computational modeling, it should be noted that the validity of the conclusions must be understood within the limits of the fit of the computational models. We used the PVL model with the decay-reinforcement learning rule because it showed the best model-fit and because previous simulation studies show relatively accurate parameter values and better parameter consistency across tasks than other competing models. However, the model's performance on long-term predictions appears worse than other model such as the PVL model with the delta rule. Future studies should focus on developing models that have both good short-term and long-term prediction accuracy. Finally, study findings are limited to the IGT, which was our only measure of decision-making; therefore, these findings need to be examined further with other decision-making tasks.

In summary, this study extends findings with drug users to a new and relatively understudied population of middle-aged women with a history of crack cocaine and/or heroin use and is the first to apply a formal mathematical model to examine the effects of HIV on cognitive, motivational, and affective processes involved in complex decision-making. Our findings underscore the potential importance of using performance indices such as computational modeling parameters, which may be more sensitive for revealing the underlying cognitive and motivational decision-making biases in different clinical populations than standard indices of overt behavioral performance. The current study contributes to a growing line of literature which shows that different psychological processes may underlie similar overt behavioral performance in various clinical groups and may be associated with different functional outcomes.

## Supporting Information

Figure S1 **Parameter estimates of A (learning/memory) after controlling for age.** Note: 300 random samples were drawn from the posterior distributions for each group. Dashed lines indicate mean values for each group. HDI = mean and 95% HDI range.
(TIF)

Figure S2 **Parameter estimates of λ (loss aversion) after controlling for age.** Note: 300 random samples were drawn from the posterior distributions for each group. Dashed lines indicate mean values for each group. HDI = mean and 95% HDI range.
(TIF)

File S1 **Appendix S1, Model Comparisons. Appendix S2, Prospect Valence Learning Model with the decay-reinforcement learning rule. Appendix S3, Hierarchical Bayesian estimation.**
(DOC)
