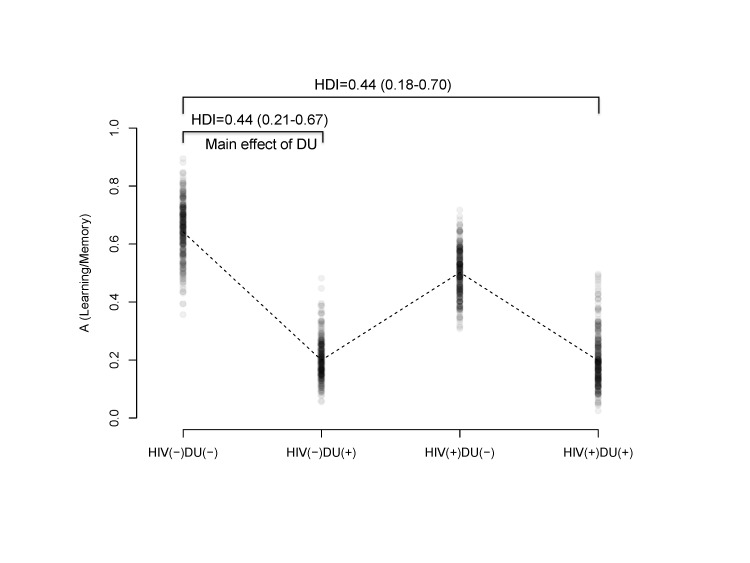# Correction: Computational Modeling Reveals Distinct Effects of HIV and History of Drug Use on Decision-Making Processes in Women

**DOI:** 10.1371/annotation/5a8e6fe0-623c-4d17-8781-9a0eadf67a43

**Published:** 2013-09-10

**Authors:** Jasmin Vassileva, Woo-Young Ahn, Kathleen M. Weber, Jerome R. Busemeyer, Julie C. Stout, Raul Gonzalez, Mardge H. Cohen

There was an error in Figure 2. The correct version of the figure is available here: 

**Figure:**