# Supplementary figures and images for: Computational Modeling Reveals Distinct Effects of HIV and History of Drug Use on Decision-Making Processes in Women
Source: PLoS One. 2013 Aug 7;8(8):e68962. doi: 10.1371/journal.pone.0068962 (PMC3737214; doi:10.1371/journal.pone.0068962)

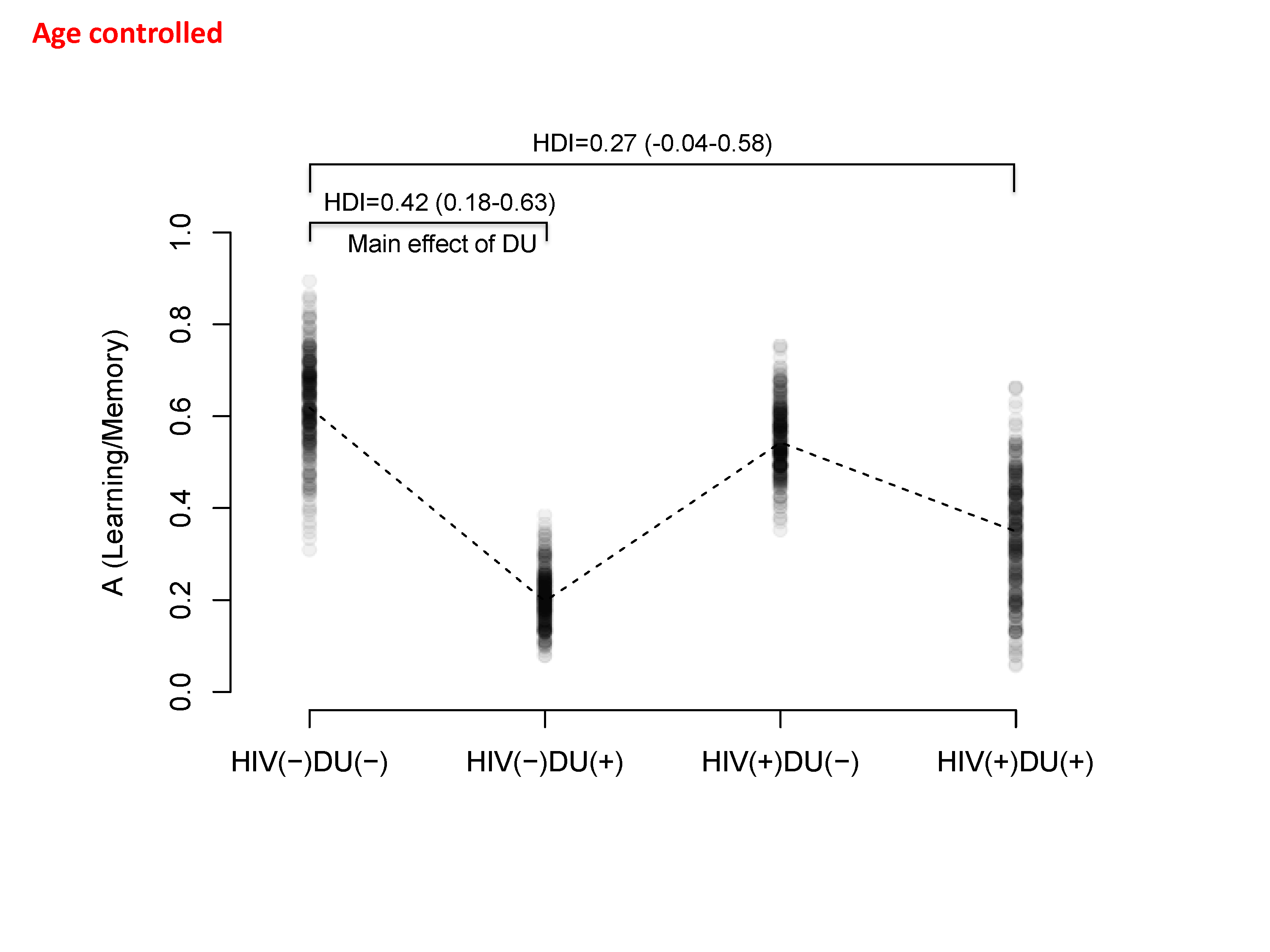

Supplement: Figure S1 — Parameter estimates of A (learning/memory) after controlling for age. Note: 300 random samples were drawn from the posterior distributions for each group. Dashed lines indicate mean values for each group. HDI = mean and 95% HDI range. (TIF) [file pone.0068962.s001.tif]

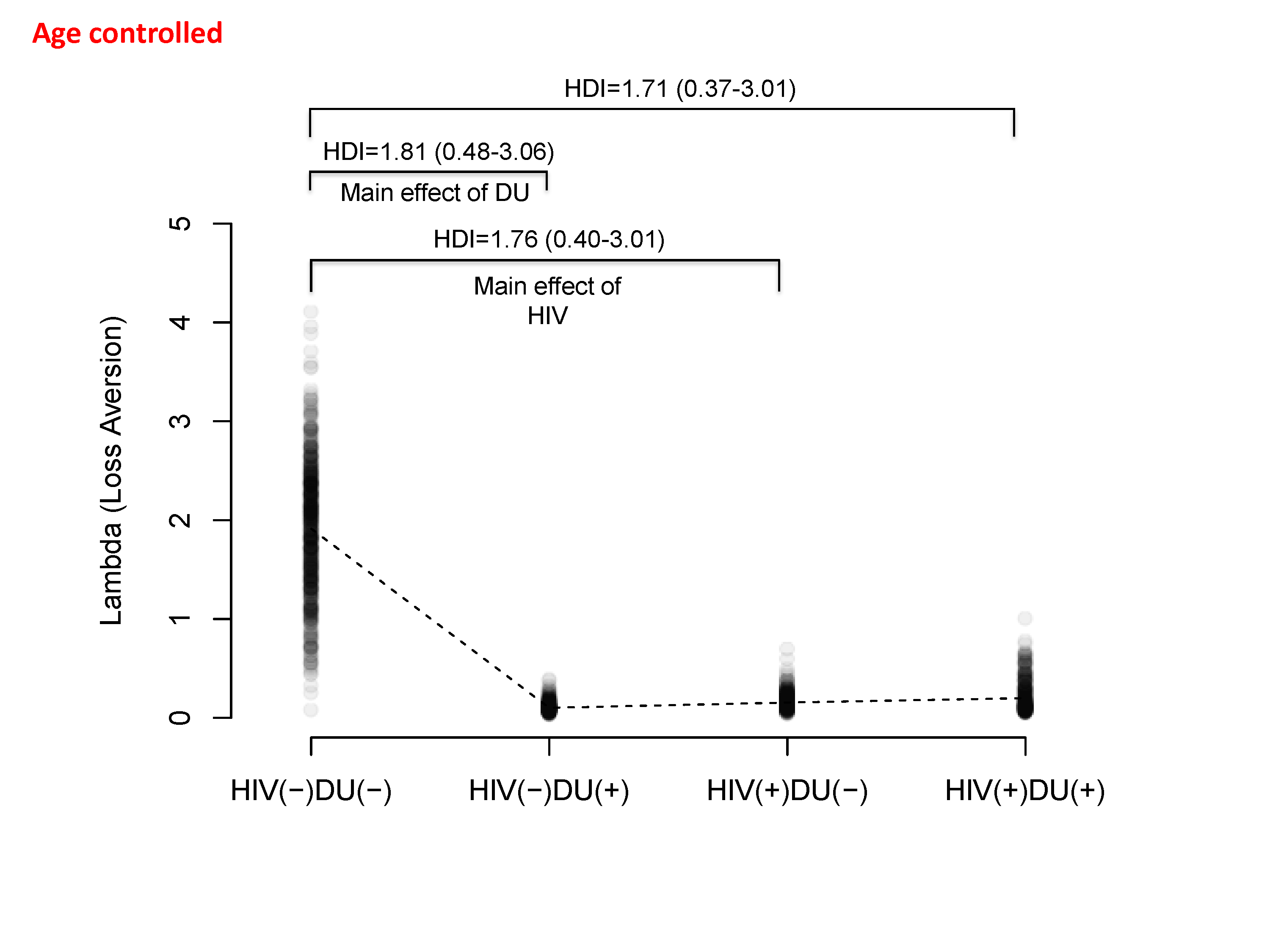

Supplement: Figure S2 — Parameter estimates of λ (loss aversion) after controlling for age. Note: 300 random samples were drawn from the posterior distributions for each group. Dashed lines indicate mean values for each group. HDI = mean and 95% HDI range. (TIF) [file pone.0068962.s002.tif]
