# Supplementary material for: Computational Modeling Reveals Distinct Effects of HIV and History of Drug Use on Decision-Making Processes in Women
Source: PLoS One. 2013 Aug 7;8(8):e68962. doi: 10.1371/journal.pone.0068962 (PMC3737214; doi:10.1371/journal.pone.0068962)
Supplement: File S1 — Appendix S1, Model Comparisons. Appendix S2, Prospect Valence Learning Model with the decay-reinforcement learning rule. Appendix S3, Hierarchical Bayesian estimation. (DOC) [file pone.0068962.s003.doc]

**Supporting Information**

**Appendix S1. Model Comparisons**

The first step in computational modeling is to compare and test competing models, which typically vary in terms of some of their basic rules (Busemeyer & Diederich, 2010). The *learning rules* of various decision-making models differ according to the manner in which past expectancies are updated based on new feedback, such that in *interference models* only the chosen option is updated, whereas in *decay models* the chosen option is updated and the unchosen options are discounted by a certain amount (Yechiam & Busemeyer, 2005). The *choice rules* of different models also differ based on the way in which they map past experience onto choice behavior, with some models choosing always the option producing the maximum expectation, whereas in others choices are probabilistically determined based on the strength of the expectation (Yechiam & Busemeyer, 2005).

The first model that we tested was the Expectancy-Valence Learning (EVL) model (Busemeyer & Stout, 2002), one of the most widely used computational models of decision-making for the IGT, which assumes that the gains and losses experienced after making a choice produce an affective reaction called a valence. The model includes 3 parameters representing different component functions of decision-making: (1) *Motivational parameter*, manifested by greater attention weight given to gains versus losses, (2) *Cognitive (learning/memory) parameter* captured by the tendency to remember more recent events and forget past outcomes, and (3) *Response sensitivity parameter*, measuring consistency of choices or tendency to respond randomly or impulsively (Busemeyer & Stout, 2002; Lane et al., 2006; Yechiam, Busemeyer, Stout & Bechara, 2005). As detailed below, we compared the EVL model to two variants of the more recently developed Prospect Valence Learning (PVL) model (Ahn et al., 2008), which use a nonlinear utility function such as that used in prospect theory (Tversky & Kahneman, 1992).

To determine which computational model best fits our data, we used maximum likelihood estimation (MLE) methods to fit the model to each person separately, and then used a Bayesian information criterion to compare the Bernoulli baseline against: (1) the “classic” EVL model (Busemeyer & Stout, 2002); (2) the PVL model with the delta learning rule , a type of *interference model* which assumes that memory is changed by relevant events and not simply as a function of time; and (3) the PVL model with the decay-reinforcement learning rule , representative of *decay models*, which assume that memory decay occurs purely as a function of time and without the occurrence of interfering events. This rule has more flexibility than the delta rule because it permits the expectancies of all alternatives to change on each trial (Yechiam & Busemeyer, 2005). The BIC score is a statistic that combines badness of fit with a penalty for number of parameters. To evaluate the models, we used a BIC change score that measures the improvement of the computational model over the baseline model (BIC change equals the BIC from the baseline model minus the BIC from the computational model). Therefore positive BIC changes represent improvement over baseline and accordingly the model with the highest BIC change is considered the best. The mean (and standard deviation) of BIC change scores across all participants were 1.85 (20.9), 4.73 (19.6), and 16.75 (29.6) for the EVL model, the PVL with the delta learning rule, and the PVL with the decay-reinforcement learning rule, respectively. Note that higher BIC indicates a better model fit. When tested across all participants, non-parametric signed rank tests revealed that the PVL model with the decay-reinforcement learning rule had better model-fits than the PVL model with the delta learning rule (*p* < .001) and the EVL model (*p* < .001). This is in line with the literature showing that the PVL model provides better model fits, simulation performance and generalizability than the EVL model .

**Appendix S2. Prospect Valence Learning Model with the decay-reinforcement learning rule**

The model has three components. First, the outcome evaluation follows the Prospect utility function which has diminishing sensitivity to increases in magnitude and different sensitivity to losses versus gains (i.e., loss aversion). The utility, *u(t)* on trial *t* of each net outcome *x(t)* is expressed as:


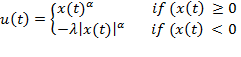
 (1)

Here *α* (shape parameter, 0 < *α* < 1) governs the shape of the utility function and *λ* (loss aversion parameter, 0 < *λ* < 5) determines the sensitivity of losses compared to gains. Net outcomes were scaled for computational modeling so that the median highest net gain across subjects in the first block of ten trials becomes 1 and the largest net loss becomes -11.5 . A value of loss aversion (*λ*) greater than 1 indicates that the individual is more sensitive to losses than to gains. A value of *λ* less than 1 indicates that the individual is more sensitive to gains than to losses.

Based on the outcome of the chosen option, the expectancies of decks were computed using the decay-reinforcement learning rule . In the decay-reinforcement learning rule, the expectancies of all decks are discounted with each trial and then the current outcome utility is added to the expectancy of the chosen deck:


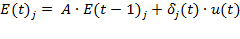
 (2)

*A* (recency parameter, 0 < *A* < 1) determines how much the past expectancy is discounted. *δ_j_(t)* is a dummy variable which is 1 if deck *j* is chosen and 0 otherwise. The softmax choice rule was then used to compute the probability of choosing each deck *j*. *θ(t)* (sensitivity) governs the degree of exploitation versus exploration:


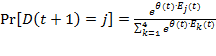
 (3)

*θ(t)* is assumed to be trial-independent and was set to 3^c^ - 1 . *c* is the *consistency* parameter (choice sensitivity) and was limited from 0 to 5 so that the sensitivity ranges from 0 (random) to 242 (almost deterministic).

### Appendix S3. Hierarchical Bayesian estimation

We used hierarchical Bayesian analysis (HBA) to estimate model parameters. For more details on HBA, there are some excellent review papers (Lee, 2008; Lee, 2011; Shiffrin et al., 2008).

In our formation of the HBA, it was assumed that the parameters of individual participants follow parent distributions, which were modeled with independent truncated normal distributions for each parameter:


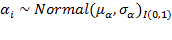
,


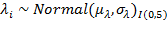
,


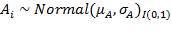
, (4)


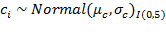
.

μ_x_ and σ_x_ indicate the mean and the standard deviation of the normal distribution of each group-level parameter *x*. *α* and *A* were limited to values between 0 and 1, and *λ* and *c* were limited to values between 0 and 5. For the prior distributions of the parameters, the uniform distributions were used for μ_x_, and Gamma(.001, .001) for precisions (=1/ σ_x_^2^), which are weakly informative priors.

OpenBUGS was used for Markov chain Monte Carlo (MCMC) sampling and for posterior inference. A total of 100,000 samples were drawn after 100,000 burn-in samples with three chains. For each parameter, the Gelman-Rubin test was run to confirm the convergence of the chains (a.k.a. Rhat). Rhat values, which show the convergence of parameters, were 1.00 for almost all parameters, and at most 1.04, which suggested MCMC chains converged to the target posterior distributions.

**Hierarchical Bayesian Analysis Controlling For Age**

We conducted additional HBA parameter estimation controlling for age for the parameters on which we observed group differences (*A* and ). Specifically, and in Equation (4) were modified as follows:

Here, and are the age-controlled and parameters of Equation (1). and are parameters assessing the effect of z-scored ages (Z_age_). Z_age_ were computed across all participants and the parameters were estimated separately for each group. We used identical formulation of the HBA, except that the normal distribution with a mean=0 and a SD=10^6^ was used as a prior distribution for and . Consistent with results without controlling for age, we found a main effect of DU on A and and a main effect of HIV on (see Figures A1 and A2 for details).

**References**

Ahn, W.-Y., Busemeyer, J. R., Wagenmakers, E.-J., & Stout, J. C. (2008). Comparison of decision learning models using the generalization criterion method. *Cognitive Science, 32*, 1376-1402. doi: 10.1080/03640210802352992.

Busemeyer, J.R., & Diederich, A. (2010). *Cognitive modeling.* Thousand Oaks, CA: SAGE Publications.

Busemeyer, J. R., & Stout, J. C. (2002). A contribution of cognitive decision models to clinical assessment: Decomposing performance on the Bechara gambling task. *Psychological Assessment, 14(3)*, 253-262. doi: 10.1037/1040-3590.14.3.253

Erev, I., & Roth, A. (1998). Predicting how people play games: Reinforcement learning in experimental games with unique, mixed strategy equilibria. *American economic review*, *88(4)*, 848-881.

Fridberg, D. J., Queller, S., Ahn, W.-Y., Kim, W., Bishara, A. J., Busemeyer, J. R., . . . Stout, J. C. (2010). Cognitive mechanisms underlying risky decision-making in chronic cannabis users. *Journal of Mathematical Psychology, 54*(1), 28-38. doi: 10.1016/j.jmp.2009.10.002

Gelman, A., Carlin, J. B., Stern, H. S., & Rubin, D. B. (2004). *Bayesian Data Analysis (2nd ed.)*: Chapman & Hall/CRC.

Lane, S. D., Yechiam, E., & Busemeyer, J. R. (2006). Application of a computational decision model to examine acute drug effects on human risk taking. *Experimental and Clinical Psychopharmacology, 14(2)*, 254-264. doi: 10.1016/s0028-3932(00)00136-6

Lee, M. (2008). Three case studies in the Bayesian analysis of cognitive models. *Psychonomic Bulletin and Review, 15(1)*, 1-15.

Lee, M. (2011). How cognitive modeling can benefit from hierarchical bayesian models. *Journal of Mathematical Psychology, 55(1)*, 1-7. doi:10.1016/j.jmp.2010.08.013

Rescorla, R. A., & Wagner, A. R. (1972). *A theory of Pavlovian conditioning: variations in the effectiveness of reinforcement and nonreinforcement*. In A.H. Black & W.F. Prokasy (Eds.), Classical conditioning II:current research and theory (pp. 64-99) New York, NY: Appleton-Century-Crofts.

Rouder, J. N., & Lu, J. (2005). An Introduction to Bayesian Hierarchical Models with an Application in the Theory of Signal Detection. *Psychonomic Bulletin Review, 12(4)*, 573-604.

Rouder, J. N., Lu, J., Speckman, P. L., Sun, D., & Jiang, Y. (2005). A Hierarchical Model for Estimating Response Time Distributions. *Psychonomic Bulletin &amp; Review, 12(2)*,195-223

Schwartz, G. (1978). Estimating the dimension of a model. *Annals of Statistics, 5*, 461-464.

Shiffrin, R., Lee, M., Kim, W., & Wagenmakers, E. (2008). A Survey of Model Evaluation Approaches With a Tutorial on Hierarchical Bayesian Methods. *Cognitive Science, 32(8)*, 1248-1284. doi:10.1080/03640210802414826

Thomas, A., O'Hara, B., Ligges, U., and Sturtz, S. (2006). Making BUGS Open. R News 6 (1), 12-17.

Tversky, A., & Kahneman, D. (1992). Advances in prospect theory: Cumulative representation of uncertainty. Journal of Risk and Uncertainty, *5 (4)*, 297-323.

Wetzels, R., Vandekerckhove, J., Tuerlinckx, F., & Wagenmakers, E. (2010). Bayesian parameter estimation in the Expectancy Valence model of the Iowa gambling task. *Journal of Mathematical Psychology, 54*, 14-27.

Yechiam, E., & Busemeyer, J. R. (2005). Comparison of basic assumptions embedded in learning models for experience-based decision making. *Psychonomic Bulletin & Review, 12(3)*, 387-402. doi: 10.3758/BF03193783

Yechiam, E., Busemeyer, J. R., Stout, J. C., & Bechara, A. (2005). Using cognitive models to map relations between neuropsychological disorders and human decision-making deficits. *Psychological Science, 16*(12), 973-978. doi:10.1111/j.1467-9280.2005.01646.x
